# Linked nosocomial COVID-19 outbreak in three facilities for people with intellectual and developmental disabilities due to SARS-CoV-2 variant B.1.1.519 with spike mutation T478K in the Netherlands

**DOI:** 10.1186/s12879-022-07121-y

**Published:** 2022-02-10

**Authors:** Koen M. F. Gorgels, Jozef Dingemans, Brian M. J. W. van der Veer, Volker Hackert, Audrey Y. J. Hensels, Casper D. J. den Heijer, Lieke B. van Alphen, Paul H. M. Savelkoul, Christian J. P. A. Hoebe

**Affiliations:** 1 Department of Sexual Health, Infectious Diseases and Environmental Health, South Limburg Public Health Service, PO Box 33, 6400 AA Heerlen, The Netherlands; 2 Department of Medical Microbiology, Care and Public Health Research Institute (CAPHRI), Faculty of Health, Medicine and Life Sciences, Maastricht University Medical Centre (MUMC+), PO Box 5800, 6202 AZ Maastricht, The Netherlands; 3 Department of Social Medicine, Care and Public Health Research Institute (CAPHRI), Faculty of Health, Medicine and Life Sciences, Maastricht University, PO Box 616, 6200 MD Maastricht, The Netherlands

**Keywords:** SARS-CoV-2, Outbreak, Intellectual and developmental disability, B.1.1.519 variant, T478K spike mutation, Infection prevention and control

## Abstract

**Background:**

Individuals with intellectual and developmental disabilities (IDD) living in congregated settings have increased risk of COVID-19 infection and mortality. Little is known about variant B.1.1.519 with spike mutation T478K, dominant in Mexico. We describe a linked SARS-CoV-2 B.1.1.519 outbreak in three IDD facilities in the Netherlands.

**Methods:**

Following notification of the index, subsequent cases were identified through serial PCR group testing. Positive specimens were submitted for whole-genome-sequencing. Clinical information was gathered through interviews with staff members of the three facilities.

**Results:**

Attack rate (AR) in clients of the index facility was 92% (23/25), total AR in clients 45% (33/73) and in staff members 24% (8/34). 55% (18/33) of client cases were asymptomatic, versus 25% (2/8) of staff members. Five client cases (15%) were hospitalized, two died (6%). Sequencing yielded the same specific B.1.1.519 genotype in all three facilities. No significant difference in median viral load was established comparing the B.1.1.519 variant with other circulating variants. The index of the linked outbreak reported no travel history or link to suspected or confirmed cases suggesting regional surveillance. Observed peak regional prevalence of B.1.1.519 during the outbreak supports this.

**Conclusion:**

AR, morbidity and mortality prior to control measures taking effect were high, probably related to the specific characteristics of the IDD setting and its clients. We assessed no evidence for intrinsic contributing properties of variant B.1.1.519. Our study argues for enhanced infection prevention protocols in the IDD setting, and prioritization of this group for vaccination against COVID-19.

**Supplementary Information:**

The online version contains supplementary material available at 10.1186/s12879-022-07121-y.

## Background

A cluster of patients with pneumonia of unknown origin, detected in the Chinese city of Wuhan in December 2019, was determined shortly after being associated with a novel coronavirus, severe acute respiratory syndrome coronavirus 2 (SARS-CoV-2) [1]. The disease, termed COVID-19, soon spread over the entire world and was declared a pandemic on 11 March, 2020 [2].

Individuals with intellectual and developmental disabilities (IDD) have increased risk for contracting COVID-19 [3] and COVID-19 related mortality [4]. In particular, living in congregated settings, like residential care facilities, increases risk of fatal outcomes [5, 6]. The World Health Organization (WHO) reports that overcrowding, lack of facilities for personal and environmental hygiene, insufficient isolation facilities and inadequate numbers of supervising staff may compromise infection control in long-stay mental care institutions [7]. Staff members may have difficulty maintaining physical distance, and revised rules and protocols may be challenging to introduce or explain. Additionally, while institutions tend to report adequate access to Personal Protective Equipment (PPE), unfamiliarity with its use may compromise adequate application and increase anxiety among staff members [7].

Random mutations leading to the rise of different variants of SARS-CoV-2 may complicate efforts to control the pandemic globally. The WHO categorizes mutations associated with certain phenotypical changes as variants ‘of interest’, while others featuring increased transmissibility, virulence or vaccine resistance are referred to as variants ‘of concern’ [8]. One of the variants labelled ‘of concern’ is B.1.1.7, which originated in the United Kingdom and has become the dominant line in the Netherlands during the 1st months of 2021 [9]. Little information is available on the B.1.1.519 variant with spike mutation T478K which became dominant in Mexico over the course of a few months: in October 2020, 5% of sequenced specimens were reported to be B.1.1.519, whereas in February 2021, this percentage had increased to 87% [10]. Spike mutation T478K lies within the interaction domain with the human receptor ACE2 [11]. Additionally, B.1.1.519 shares the P681H mutation with the B.1.1.7 variant, which is near the furin-cleavage site and could have an effect on viral entry [12].

This paper describes the first confirmed introduction of B.1.1.519 as a novel variant in the Netherlands after analysis of positive cases related to three outbreaks in facilities for people with IDD: two residential care facilities and a care farm with daycare activities. All outbreaks were linked epidemiologically, with cases from both residential care facilities attending the care farm. Moreover, the same sequence type was encountered in clients from all facilities. We report epidemiological, microbiological and sequencing findings from our investigation of the linked outbreak and subsequent outbreak control measures.

## Methods

### Epidemiological investigation

We performed a retrospective observational study in three facilities for individuals with IDD. All individuals with IDD described had mild to moderate intellectual disability. None were immunodeficient or vaccinated against COVID-19. The Netherlands initiated their vaccination program January 6, 2021. Due to a shortage of vaccines medical personnel working in acute care and nursery homes were prioritized for vaccination. Vaccination for staff members working in facilities for individuals with IDD and individuals with IDD started during February 2021. All outbreaks described were investigated by the South Limburg Public Health Service, the Netherlands. Additional information was obtained through interviews with medical and managerial staff of the affected institutions.

### Case definition

A case was defined as an individual with a real-time polymerase chain reaction (RT-PCR) test positive for SARS-CoV-2. Cases were defined as symptomatic if they reported COVID-19 related symptoms, including common cold symptoms (nasal cold, runny nose, sneezing or sore throat), cough, elevated temperature or fever (temperature > 38 °C), loss of taste or smell, diarrhea, nausea, fatigue and headache [13]. Based on Dutch guidelines [14] cases were defined as asymptomatic if they reported no symptoms at the time of their positive test and developed no symptoms in the 7 days that followed.

In symptomatic cases, the day of symptom onset was used as disease onset. In asymptomatic and pre-symptomatic cases, the date of the positive test was used as a proxy for disease onset. Cases reporting chronic symptoms, indistinguishable from symptoms of recent onset, were also classified by the date of their positive test.

Cases were further classified into two different categories, i.e., clients and staff members. Clients were defined as any person receiving care at the affected institutions, including those following daytime activities at the care farm. Staff members were defined as individuals employed by any of the three institutions.

### Testing strategy

According to national/regional outbreak management protocols for residential care facilities, all clients and staff members are tested after onset of first positive cases once weekly until no new cases are reported. All close contacts of confirmed cases are recommended to be tested twice, i.e., once as soon as possible after exposure, and again 5 days after exposure.

Oropharyngeal and nasopharyngeal swabs were collected by trained personnel and pooled together in viral transport medium (Mediaproducts, The Netherlands). All group testing was sent to the Medical Microbiological Laboratory of Maastricht University Medical Centre for analysis within 1 h after test application. Laboratory confirmation of SARS-CoV-2 was performed via an RT-PCR assay. First, for RNA extraction, 900 µl of clinical sample was mixed with 900 µl of Chemagic Viral Lysis Buffer (Perkin-Elmer) and RNA was extracted from samples using the Chemagic Viral DNA/RNA 300 Kit H96 (Perkin-Elmer) on the Chemagic 360 system (Perkin-Elmer). A multiplex RT-PCR was performed using the N1-gene and E-gene as targets, including the immediate early gene of mouse cytomegalovirus as an internal control (Additional file 1: Table S1). cDNA synthesis and PCR amplification were combined using the TaqPath™ 1-Step RT-qPCR Master Mix, CG (Applied Biosystems, US). Thermal cycling was performed using the Quantstudio 5 Real-Time PCR System (Applied Biosystems, US). Oligonucleotides were synthesised and provided by Biolegio (Netherlands) (Additional file 1: Table S1). A Kruskal–Wallis test was performed to compare the median CT-values of the B.1.1.7, B.1.1.519 and other variants, followed by a Dunn’s multiple comparison post-hoc test.

### Sequencing of SARS-CoV-2-positive samples

Samples that were tested positive for SARS-CoV-2 were stored at – 80 °C until RNA was isolated for sequencing. For RNA extraction, 90 µl of sample was mixed with 90 µl of Chemagic Viral Lysis Buffer (Perkin-Elmer), followed by extraction using the MagNA Pure 96 DNA and Viral NA Small Volume Kit 96 (Roche, Germany) on the MagNA Pure 96 system (Roche, Germany), without the addition of an internal extraction control.

Sequencing was performed using the PCR tiling of SARS-CoV-2 virus with Native Barcoding Expansion 96 (EXP-NBD196) protocol (Version: PTCN_9103_v109_revH_13Jul2020) of Oxford Nanopore technologies, with minor modifications and using the primers previously published by Oude Munnink et al. [15]. Briefly, the only modifications were extending the barcode and adaptor ligation steps up to 60 min and loading 48 samples per flow cell.

Bioinformatic analysis was performed using an in-house developed pipeline MACOVID that is based on Artic v1.1.3. In brief, short and obvious chimeric reads are filtered with Cutadapt v2.5. The filtered reads were mapped to the reference genome MN908947.3 with Minimap2 v2.17 and quality checked with “align_trim” function of Artic v1.1.3. Mapped reads were split per primer pool using Samtools v1.9 and a consensus was created per primer pool with Medaka v1.0.3. Variants were called using Medaka v1.0.3 and Longshot v0.4.1. Low coverage regions (< 30×) were masked with “artic_make_depth_mask” function of Artic v1.1.3. A preconsensus was made with “artic_mask” and the final consensus sequence was made with bcftools v1.10.2. Documentation and source code are available from https://github.com/MUMC-MEDMIC/MACOVID under MIT license. The consensus sequences were used to construct a phylogenetic tree with ncov pipeline v3 of nextstrain with all B.1.1.519 and Dutch genomes in the Global Initiative on Sharing All Influenza Data (GISAID) (15-Apr-2021) as a reference.

### Medical ethical approval

In the Netherlands, research is required to undergo review by an accredited Medical Research Ethics Committee if it is subject to the Dutch Medical Research Involving Human Subjects Act (WMO). Retrospective research (that is carried out on existing patient material and/or existing patient files) is exempt from the WMO, according to the Dutch Central Committee on Research Involving Human Subjects (CCMO) [16]. All data presented in this paper, including information obtained from affected institutions, were retrospectively retrieved from regular infectious disease control activities and were de-identified. As such, our study does not fall under the scope of the WMO and therefore is exempt from medical ethical approval. No additional administrative permissions were required to use the data as it is owned by the South Limburg Public Health Service.

## Results

### Outbreak 1: residential care facility 1

Outbreak 1 occurred in a residential care facility which houses 25 clients. Each client had their own room with private sanitation. Two had Down syndrome. A full range of infection prevention measures to manage the risk related to Covid-19 transmission had been implemented, including the use of medical face masks by staff members. Staff comprised 15 members who all worked before and during the outbreak.

Four clients tested positive on 16 February, 2021 after multiple clients reported symptoms. One case had visited the care farm for daytime activities on 12 February, probably being the index for outbreak 2. Source tracing revealed a family member of one client as a putative source. This individual reported onset of symptoms on 5 February and tested positive on 9 February.

### Outbreak control measures

Following identification of these four cases, additional control measures to prevent further transmission were implemented. Individual room isolation was mandated for all clients, a visitor ban was declared, temperature was monitored twice daily for all clients, and staff members were instructed to change PPE equipment every 3h while working on the location. PPE consisted of FFP2/N95 face masks, gloves, goggles and gown.

The first group testing session, comprising the 21 hitherto untested clients, was performed on 18 February and resulted in 15 cases positive for SARS-CoV-2. Of these, 14 reported no symptoms on the day of testing, but four developed symptoms over subsequent days. All staff members were tested as well, one of whom tested positive. Four staff members and two clients tested positive the days after.

A second group testing session was performed on 25 February on the four remaining clients and staff members who had tested negative before yielding two new client cases. A third group testing session was conducted on 4 March yielding no new cases.

### Outbreak 2: a care farm

Outbreak 2 appeared in a care farm which offers daycare activities for 35 clients. All daycare clients live at residential care facilities or at home with their families. The care farm employs 10 staff members. Activities take place in groups of 8 clients at most, physical distance between clients and staff members is maintained and supervised during activities, and the use of non-medical face masks is mandatory for clients and staff members.

As mentioned earlier, a client from outbreak 1 had visited the care farm on 12 February. This client, who had a chronic cough but no other symptoms, tested positive on 16 February. One other day care farm client developed symptoms on 14 February and also tested positive on 16 February. This case inhabits residential care facility 2, probably being the index for outbreak 3.

### Outbreak control measures

Following notification of these two cases, the care farm closed its daycare activities on 17 February. Two exposed clients tested positive on 18 February. Three clients and two staff members developed symptoms and tested positive in the following days.

Group testing was performed on all clients and staff members on 20 February, yielding one client case. On 26 February, a second round of group testing on all staff members and 14 clients revealed one more positive client. No further group testing sessions were carried out.

### Outbreak 3: residential care facility 2

Outbreak 3 occurred in residential care facility 2 which houses 15 clients. The residential setting and implemented infection prevention measures were similar as described in outbreak 1. Staff comprised nine members who all worked before and during the outbreak.

The client case, that had visited the care farm case, reported symptoms from 14 February and was immediately put into isolation, followed by a positive test on 16 February.

### Outbreak control measures

Similar outbreak prevention measures were implemented as in outbreak 1. Staff members were tested yielding one asymptomatic case on 20 February. Group testing on clients, performed on 22 February, yielded two cases. Restraints were lifted for all clients who tested negative. No further group testing sessions were carried out.

### Summary

**Fig. 1:**
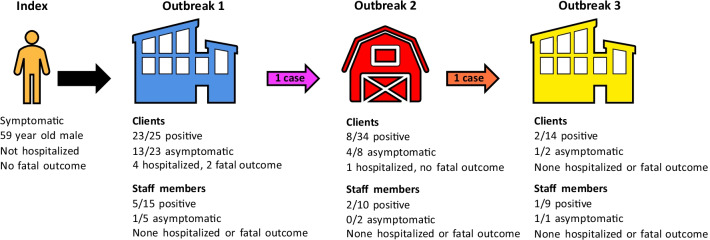
Schematic reconstruction of the three reported outbreaks. All cases among clients and staff members are summarized. For calculation of the attack rate (AR) all clients and staff members at risk were calculated

**Fig. 2:**
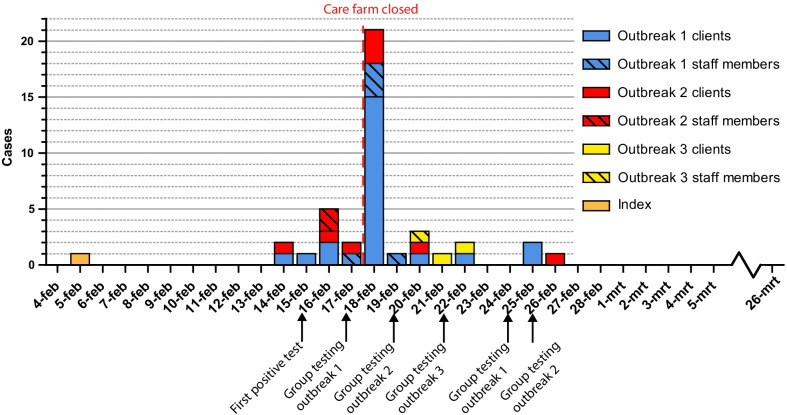
Chronological reconstruction of the three reported outbreaks, based on date of symptom onset or date of positive test

Figure 1 shows a probable schematic reconstruction and Fig. 2 shows a chronological reconstruction of the three outbreaks. In outbreak 1 an attack rate (AR) of 92% was observed among clients. In outbreak 2 and outbreak 3 the AR was 24% and 14% respectively. Client cases were asymptomatic in 55% (18/33) of cases versus 25% (2/8) in staff members. Among client cases, 5 were hospitalized and two died (case mortality rate 6%). During the 1-month follow-up five additional cases among family members of clients and staff members were identified and epidemiologically linked to the outbreaks.

### Sequence analysis

The cycle threshold (Ct) of the RT-PCR test of one of the pre-symptomatic clients who tested positive on 18 February was 8. This client suffered from diabetes mellitus and obesity and was hospitalized later. Because of the high viral load, the sample obtained from this subject was selected for SARS-CoV-2 sequence analysis. The SARS-CoV-2 genotype in this patient belonged to the B.1.1.519 lineage. Subsequently, sequencing was performed on samples from all clients and staff members from all outbreaks and the family member suspected of being the putative source.

**Fig. 3:**
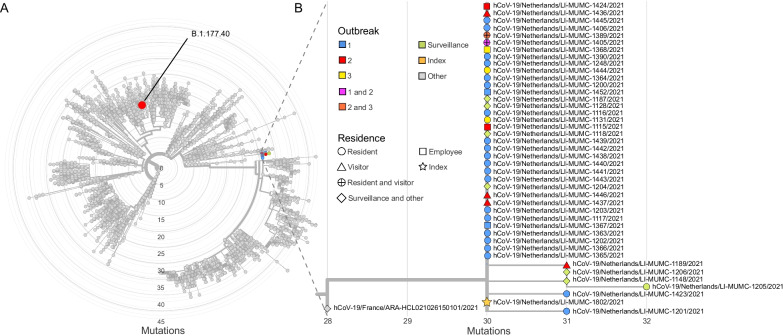
Phylogenetic relationship between the isolates in this study. **A** Overview of the position of all isolates belonging to the B.1.1.519 lineage and one isolate belonging to the B.1.177.40 lineage in the phylogenetic tree. **B** Zoomed-in portion of the B.1.1.519 lineage highlighting all residues belonging to outbreak 1, outbreak 2, cluster 3 as well as the index. The closest ancestor is highlighted in gray

Of the 42 samples obtained, 36 were successfully sequenced. Thirty-five isolates belonged to the B.1.1.519 lineage (32 sequences being identical, while three sequences harbored one additional mutation), whereas one SARS-CoV-2 isolate from a client of outbreak 2, belonged to the B.1.177.40 lineage (Fig. 3). These findings linked all outbreaks and indicated that a family member was the index of the linked outbreak. The index was subsequently contacted and reported no travel history or link to other confirmed or suspected cases.

### Viral load analysis

**Fig. 4:**
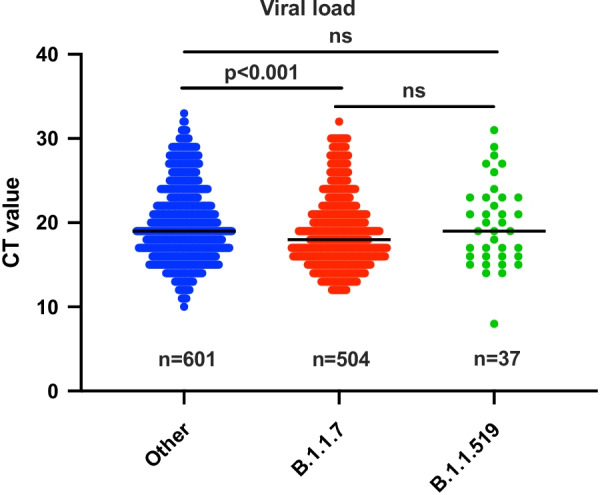
Distribution of Ct-values obtained for samples harboring isolates belonging to B.1.1.7, B.1.1.519 and other lineages. The median value is indicated by a horizontal line. *ns* not significant. Only samples harboring isolates without mutations leading to an amino acid change between positions 319 and 541 in the receptor binding domain of the spike protein were included

Since the B1.1.519 lineage harbors a few mutations of interest in the spike protein including the T478K mutation (Additional file 1: Figure S1) and the P681H mutation as established in several other lineages, including the B.1.1.7 variant [12], we studied whether samples harboring this genotype contained higher viral loads compared to isolates belonging to the B.1.1.7 or other variants. To exclude the effect of other mutations in the receptor-binding domain in isolates belonging to the B.1.1.7 lineage or other variants, we removed any isolates harboring a mutation that leads to an amino acid change between positions 319 and 541 in the spike protein from the analysis. A significant difference was only determined when comparing median Ct values of samples harboring the B.1.1.7 variant with other variants (18 versus 19, p < 0.001) (Fig. 4).

### Regional prevalence circulating genotypes

**Fig. 5:**
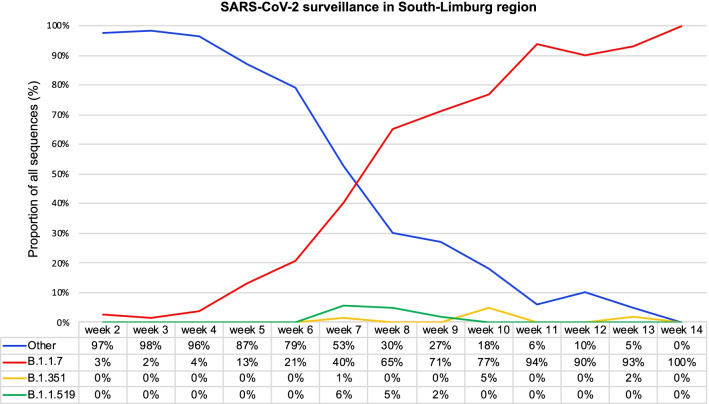
Change in prevalence of the B.1.1.7, B1.351, B.1.1.519 and other lineages in the South-Limburg region from week 2 to week 14 of 20

Since the 2nd week of 2021, SARS-CoV-2 sequencing has been performed on a weekly base to monitor the prevalence of circulating genotypes in the region of South-Limburg, as part of the national surveillance program. All SARS-CoV-2-positive cases belonging to the reported outbreaks in this study were detected in week 7 and 8, which coincides with peak prevalence of B.1.1.519 in the community (6% for week 7 and 5% for week 8) (Fig. 5). Before week 7, B.1.1.519 isolates had not been detected in the region of South-Limburg. Furthermore, the prevalence of B.1.1.519 declined gradually during week 8, after eventually disappearing in week 9. There was a strong increase in the prevalence of the B.1.1.7 lineage from week 6 (21%), over week 7 (40%) to week 8 (65%).

## Discussion

In this paper we describe a linked outbreak of B.1.1.519 with spike mutation 478 K, a novel variant of SARS-CoV-2 that is dominant mainly in Mexico [10, 11], in clients and staff members of three facilities for individuals with IDD. The linked outbreak featured a high attack rate among clients in one facility, a large proportion of asymptomatic infections, and a high case hospitalization and mortality rate. To our knowledge, this is the first report of an outbreak of SARS-CoV-2 variant B.1.1.519 in facilities for individuals with IDD.

The attack rate of 92% among clients in outbreak 1 was very high. An earlier study (publication forthcoming) conducted by South Limburg Public Health Service in residential care facilities for people with IDD describes multiple barriers in infection prevention and control. At an individual level, a high prevalence of behavioral problems, difficulties in teaching and instructing clients, lack of hygiene awareness and low risk perception may play an important role. Low health awareness in clients may hamper self-monitoring and self-reporting, especially with regard to subjective or subtle symptoms like anosmia or sore throat. Reluctance in reporting symptoms for fear of subsequent quarantine or isolation measures may also play a role [17]. Shared meals and shared living rooms of clients further facilitate transmission. Additionally, care workers in these facilities sometimes lack specific training, especially with regard to infection prevention and control. A COVID-19 serology study, performed by South Limburg Public Health Service in November 2020 (unpublished) among 10.001 inhabitants, including 1567 health care workers, showed that seroprevalence for antibodies to SARS-CoV-2 was highest in health care workers in facilities for individuals with IDD (37.1%; n = 167), compared to hospital health care workers (26.5%; n = 234) and the general population on average (19.5%). Results from both studies and other relevant sources show that effective infection control in these settings is challenging [7].

Asymptomatic and mild symptomatic infections play an important role in Covid-19 transmission and may have facilitated spread among clients and staff members in outbreak 1 [18]. The high viral load, corresponding to an unusually low Ct value, observed in one client who was pre-symptomatic at the time of testing may have facilitated rapid transmission. We determined that 55% of clients were asymptomatic compared to 25% in staff members, lending further weight to our claim that people with IDD underreport symptoms.

Variants that predominate are more likely to be associated with higher infectiousness [19]. Increased infectiousness of the B.1.1.519 variant could explain the high prevalence in Mexico and rapid transmission in our linked outbreak and could be caused by the T478K mutation or P681H which is shared with the B.1.1.7 variant. However, the B.1.1.7 variant rapidly became the dominant variant in the Netherlands, rising from 7% prevalence in early January 2021 to more than 90% by April, based on national surveillance by the National Institute for Public Health & the Environment [9]. Similar rapid rise in prevalence was observed in the South-Limburg region. The rapid surge of B.1.1.7 in the Netherlands and elsewhere, compared to a slower rise of B.1.1.519 in Mexico, may suggest that intrinsic transmissibility of B.1.1.519 is less outspoken, arguing in favor of our hypothesis that factors specific to facilities for patients with IDD played a decisive role in this outbreak.

This is further supported by our study in which the B.1.1.7 variant, but not B.1.1.519 showed a significantly lower median Ct value compared to other variants.

Super-spreading events may be a prerequisite factor in promoting widespread circulation (next to intrinsic viral transmissibility) and permanence of a novel variant in a population [19]. While B.1.1.519 did not gain a significant foothold in the Netherlands, outbreaks in residential care facilities may act as potential super-spreaders for new variants, given the specific vulnerable population of patients with IDD, the high AR, the high viral load in one case, and clients from different facilities visiting the same daycare facilities. Outbreak 3 may serve to illustrate that widespread intramural transmission can quickly be halted if rigorous infection control measures are implemented at the earliest possible stage. Our findings argue for enhanced surveillance and infection prevention and control in these settings. Likewise, institutionalized individuals with IDD should be eligible for prioritized vaccination against COVID-19.

This is also underlined by the high case fatality and hospitalization rate observed in our study. Multiple studies report intellectual disability to be a strong independent risk factor for developing COVID-19, hospitalization and mortality due to COVID-19 [4, 5]. Being diagnosed with Down syndrome in particular carries increased mortality [20].

The index of the linked outbreak reported no travel history or link to suspected or confirmed cases suggesting regional circulation of variant B.1.1.519. Weekly surveillance lends support to this hypothesis as our linked outbreak coincided with peak prevalence of the B.1.1.519. Its closest ancestor is an isolate from the Auvergne-Rhône-Alpes region in France, collected on February 9, featuring two mutations less than the majority of isolates in this study.

A strength of this study is the comprehensive data collection based on extensive interviews with staff members. Moreover, that all testing was performed using RT-PCR resulted in an highly sensitive analysis of the AR and transmissibility. Furthermore, the majority of all cases were confirmed via whole-genome sequencing of SARS-CoV-2, which is a powerful tool for studying transmission events during this stage of the pandemic.

Our study has several limitations. Due to its retrospective character findings regarding signs and symptoms of disease and days of onset may have been affected by response bias and recall bias which could have played a role in data collection. However, most data was extracted from patient files, based on observations by staff members. In outbreak 2 not all clients were included in the second round of group testing possibly leading to an underestimation of the AR. Lastly, in asymptomatic and pre-symptomatic cases the date of the positive test was used as a proxy for disease onset which may have influenced the chronological reconstruction of the outbreak.

## Conclusion and recommendations

We report the first occurrence and outbreak of SARS-CoV-2 variant B.1.1.519 in the Netherlands in three facilities for patients with IDD:

Findings from our study suggest that high attack rates, morbidity and mortality may have been related to the specific characteristics of this group of patients. We found no evidence intrinsic properties of variant B.1.1.519 contributed. Our findings argue for enhanced surveillance and infection prevention and control in these settings, and suggest that rapid implementation of rigorous infection control measures may prevent widespread intramural transmission. Our study further supports prioritization of individuals with IDD for vaccination against COVID-19.

## Supplementary Information

**Additional file 1: Figure S1. **Position of the T478K amino acid substitution in the 3D-model of the SARS-CoV-2 Spike protein of the B.1.1.519 variant in down conformation (A), or bound to the human Ace2 receptor (B). **Table S1. **Oligonucleotides used in this study.

**Additional file 2: Table S2. **GISAID accession ID’s for each described genotype

## Data Availability

Nucleic acid sequence data has been shared with the Global Initiative on Sharing All Influenza Data (GISAID) database. In the additional file an overview of all samples is provided with corresponding accession numbers.

## Abbreviations

IDD: Intellectual and developmental disabilities
PPE: Personal Protective Equipment
COVID-19: Coronavirus
SARS-COV-2: Severe acute respiratory syndrome coronavirus 2
RT-PCR: Real-time polymerase chain reaction
AR: Attack rate
Ct: Cycle threshold
WMO: Dutch Medical Research Involving Human Subjects Act
GISAID: Global Initiative on Sharing All Influenza Data
