# Supplementary material for: Linked nosocomial COVID-19 outbreak in three facilities for people with intellectual and developmental disabilities due to SARS-CoV-2 variant B.1.1.519 with spike mutation T478K in the Netherlands
Source: BMC Infect Dis. 2022 Feb 10;22:139. doi: 10.1186/s12879-022-07121-y (PMC8830120; doi:10.1186/s12879-022-07121-y)
Supplement: Supplementary file 1 — Additional file 1: Figure S1. Position of the T478K amino acid substitution in the 3D-model of the SARS-CoV-2 Spike protein of the B.1.1.519 variant in down conformation (A), or bound to the human Ace2 receptor (B). Table S1. Oligonucleotides used in this study. [file 12879_2022_7121_MOESM1_ESM.pdf]

Additional file 1

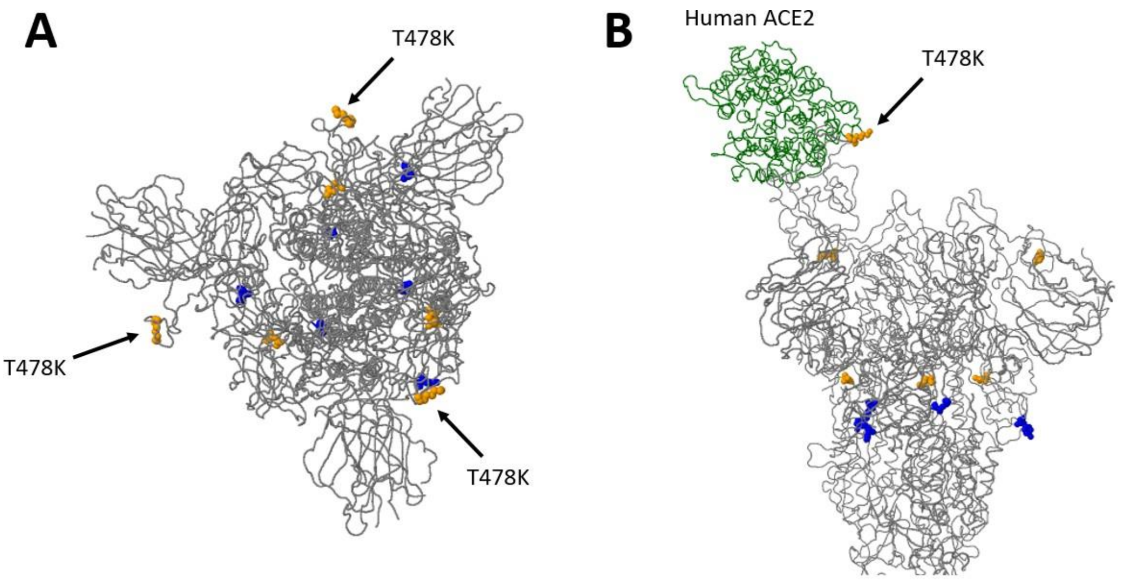

hCoV-19/Netherlands/LI-MUMC-1202/2021 (top view, down conformation)

hCoV-19/Netherlands/LI-MUMC-1202/2021 (in complex with host ACE2)

Figure S1. Position of the T478K amino acid substitution in the 3D-model of the SARS-CoV-2 Spike protein of the B.1.1.519 variant in down conformation (**A**), or bound to the human Ace2 receptor (**B**)

Table S1. Oligonucleotides used in this study.

Target	Primer/Probe	5' mod	Seq 5' – 3'	3' mod	Tm (°C)	Notes
SARS-CoV-2 E gene	SARS-CoV-2-E_F		ACAGGTACGTTAATAGTTAATAGCGT		62.6	Sarbecovirus/ SARS-CoV broad
	SARS-CoV-2-E_R		ATATTGCAGCAGTACGCACACA		64.8	
	SARS-CoV-2-E_P	FAM	ACACTAGCCATCCTTACTGCGCTTCG	BHQ1	69.3	
SARS-CoV-2 N1 gene	SARS-CoV-2-N1_F		GACCCCAAAATCAGCGAAAT		61.1	SARS-CoV-2 specific
	SARS-CoV-2-N1_R		TCTGGTTACTGCCAGTTGAATCTG		64.5	
	SARS-CoV-2-N1_P	ABY*	ACCCCGCATTACGTTTGGTGGACC	QSY	70.3	
mCMV-ie	mCMV-ie_F		CAACATTGACCACGCACTAGATG		63.6	MUMC RNA internal control
	mCMV-ie_R		TTAAACTCCCCAGGCAATGAA		62.2	
	mCMV-ie_P	Cy5	TCTTGGCCCATGCGGCACG	BHQ3	69.7	
