# Supplementary material for: Linked nosocomial COVID-19 outbreak in three facilities for people with intellectual and developmental disabilities due to SARS-CoV-2 variant B.1.1.519 with spike mutation T478K in the Netherlands
Source: BMC Infect Dis. 2022 Feb 10;22:139. doi: 10.1186/s12879-022-07121-y (PMC8830120; doi:10.1186/s12879-022-07121-y)
Supplement: Supplementary file 2 — Additional file 2: Table S2. GISAID accession ID’s for each described genotype [file 12879_2022_7121_MOESM2_ESM.docx]

**Table S2.** GISAID accession ID’s for each described genotype

| **MUMC genotype No** | **GISAID accession ID** |
| --- | --- |
| hCoV-19/Netherlands/LI-MUMC-1115/2021 | EPI_ISL_1158923 |
| hCoV-19/Netherlands/LI-MUMC-1116/2021 | EPI_ISL_1158924 |
| hCoV-19/Netherlands/LI-MUMC-1117/2021 | EPI_ISL_1158925 |
| hCoV-19/Netherlands/LI-MUMC-1118/2021 | EPI_ISL_1158926 |
| hCoV-19/Netherlands/LI-MUMC-1119/2021 | EPI_ISL_1158927 |
| hCoV-19/Netherlands/LI-MUMC-1128/2021 | EPI_ISL_1210359 |
| hCoV-19/Netherlands/LI-MUMC-1131/2021 | EPI_ISL_1210362 |
| hCoV-19/Netherlands/LI-MUMC-1148/2021 | EPI_ISL_1210378 |
| hCoV-19/Netherlands/LI-MUMC-1151/2021 | EPI_ISL_1210381 |
| hCoV-19/Netherlands/LI-MUMC-1187/2021 | EPI_ISL_1210413 |
| hCoV-19/Netherlands/LI-MUMC-1189/2021 | EPI_ISL_1210415 |
| hCoV-19/Netherlands/LI-MUMC-1200/2021 | EPI_ISL_1210426 |
| hCoV-19/Netherlands/LI-MUMC-1201/2021 | EPI_ISL_1210427 |
| hCoV-19/Netherlands/LI-MUMC-1202/2021 | EPI_ISL_1210428 |
| hCoV-19/Netherlands/LI-MUMC-1203/2021 | EPI_ISL_1210429 |
| hCoV-19/Netherlands/LI-MUMC-1204/2021 | EPI_ISL_1210430 |
| hCoV-19/Netherlands/LI-MUMC-1205/2021 | EPI_ISL_1210431 |
| hCoV-19/Netherlands/LI-MUMC-1206/2021 | EPI_ISL_1210432 |
| hCoV-19/Netherlands/LI-MUMC-1248/2021 | EPI_ISL_1210471 |
| hCoV-19/Netherlands/LI-MUMC-1363/2021 | EPI_ISL_1337836 |
| hCoV-19/Netherlands/LI-MUMC-1364/2021 | EPI_ISL_1337837 |
| hCoV-19/Netherlands/LI-MUMC-1365/2021 | EPI_ISL_1337838 |
| hCoV-19/Netherlands/LI-MUMC-1366/2021 | EPI_ISL_1337839 |
| hCoV-19/Netherlands/LI-MUMC-1367/2021 | EPI_ISL_1337840 |
| hCoV-19/Netherlands/LI-MUMC-1368/2021 | EPI_ISL_1337841 |
| hCoV-19/Netherlands/LI-MUMC-1389/2021 | EPI_ISL_1337857 |
| hCoV-19/Netherlands/LI-MUMC-1390/2021 | EPI_ISL_1337858 |
| hCoV-19/Netherlands/LI-MUMC-1405/2021 | EPI_ISL_1337872 |
| hCoV-19/Netherlands/LI-MUMC-1406/2021 | EPI_ISL_1337873 |
| hCoV-19/Netherlands/LI-MUMC-1423/2021 | EPI_ISL_1337889 |
| hCoV-19/Netherlands/LI-MUMC-1424/2021 | EPI_ISL_1337890 |
| hCoV-19/Netherlands/LI-MUMC-1436/2021 | EPI_ISL_1337900 |
| hCoV-19/Netherlands/LI-MUMC-1437/2021 | EPI_ISL_1337901 |
| hCoV-19/Netherlands/LI-MUMC-1438/2021 | EPI_ISL_1337902 |
| hCoV-19/Netherlands/LI-MUMC-1439/2021 | EPI_ISL_1337903 |
| hCoV-19/Netherlands/LI-MUMC-1440/2021 | EPI_ISL_1337904 |
| hCoV-19/Netherlands/LI-MUMC-1441/2021 | EPI_ISL_1337905 |
| hCoV-19/Netherlands/LI-MUMC-1442/2021 | EPI_ISL_1337906 |
| hCoV-19/Netherlands/LI-MUMC-1443/2021 | EPI_ISL_1337907 |
| hCoV-19/Netherlands/LI-MUMC-1444/2021 | EPI_ISL_1337908 |
| hCoV-19/Netherlands/LI-MUMC-1445/2021 | EPI_ISL_1337909 |
| hCoV-19/Netherlands/LI-MUMC-1446/2021 | EPI_ISL_1337910 |
| hCoV-19/Netherlands/LI-MUMC-1452/2021 | EPI_ISL_1337914 |
